# Clear Aligners and Smart Eye Tracking Technology as a New Communication Strategy between Ethical and Legal Issues

**DOI:** 10.3390/life13020297

**Published:** 2023-01-20

**Authors:** Alessandra Putrino, Enrico Marinelli, Mario Raso, Valeria Calace, Simona Zaami

**Affiliations:** 1 Department of Anatomical, Histological, Forensic and Orthopedic Sciences, Sapienza University of Rome, 00161 Rome, Italy; 2 Department of Medico-Surgical Sciences and Biotechnologies, Sapienza University of Rome, 04100 Latina, Italy; 3 Department of Computer Science, Sapienza University of Rome, 00161 Rome, Italy; 4 Independent Researcher, 00167 Rome, Italy

**Keywords:** appliances, removable orthodontic, eye-tracking technology, neurosciences, forensic science, legal medicine

## Abstract

Smart eye-tracking technology (SEET) that determines visual attention using smartphones can be used to determine the aesthetic perception of different types of clear aligners. Its value as a communication and comprehension tool, in addition to the ethical and legal concerns which it entails, can be assessed. One hundred subjects (50 F, 50 M; age range 15–70) were equally distributed in non-orthodontic (A) and orthodontic (B) groups. A smartphone-based SEET app assessed their knowledge of and opinions on aligners. Subjects evaluated images of smiles not wearing aligners, with/without attachments and with straight/scalloped gingival margins, as a guided calibration step which formed the image control group. Subsequently, the subjects rated the same smiles, this time wearing aligners (experimental images group). Questionnaire data and average values for each group of patients, and images relating to fixation times and overall star scores, were analyzed using these tests: chi-square, *t*-test, Mann–Whitney U, Spearman’s rho, and Wilcoxon (*p* < 0.05). One-way ANOVA and related post-hoc tests were also applied. Orthodontic patients were found to be better informed than non-orthodontic patients. Aesthetic perception could be swayed by several factors. Attachments scored lower in aesthetic evaluation. Lips distracted attention from attachments and improved evaluations. Attachment-free aligners were better rated overall. A more thorough understanding as to the opinions, expectations and aesthetic perception of aligners can improve communication with patients. Mobile SEET is remarkably promising, although it does require a careful medicolegal risk–benefit assessments for responsible and professional use.

## 1. Introduction

The appearance of a smile plays a fundamental role in judging the attractiveness of a face [1,2,3]. Such a linkage has been extensively investigated by many scientific studies aimed at evaluating the perception of smile aesthetics based on the characteristics of the occlusion as well as on the make-up of the examining population [4,5,6,7,8,9,10,11,12]. A regular and healthy-looking smile is considered an expression of well-being and self-confidence, closely linked to a younger and more attractive appearance [13,14,15,16]. Malocclusions may negatively affect quality of life as well as psychological and physical conditions [17,18,19,20]. Great attention was paid to the perception of smile aesthetics during orthodontic therapy in order to figure out how dramatically the characteristics of the appliances affected the evaluation of both orthodontic patients and dentists, as well as the population not directly involved in orthodontic treatments [21,22,23,24,25,26]. The possibility of effectively treating malocclusions, not only with fixed multibrackets but also with removable devices known as “invisible aligners” characterized by their transparency, is not recent [21,22,23,25]. Such devices have increased the level of aesthetics in orthodontics to which the introduction of composite or ceramic aesthetic brackets had already contributed as an alternative to conventional metal braces [22,23]. The indication for the use of clear aligners has considerably evolved over the years, owing to the remarkable biomechanical evolution of the systems on which they are based. Initially, they were only used to treat simple cases (such as mild crowding or diastema closure). Today, they are also widely used for moderately to extremely complex cases, and even in some cases of non-permanent dentition [27,28]. In recent years, the field of neuroscience has played a role in such an assessment through the application of so-called “smart eye-tracking technology”. Thus, neuroscience-based approaches have shifted the assessment of the perception of occlusion and orthodontic therapies to a different level from that of subjective common questionnaires and onto the visual characteristics of various orthodontic devices [29,30,31]. Smart eye-tracking or gaze-tracking technology relies on digital oculometric measurements to assess visual attention through fixation time [32,33,34]. From the outset, gaze-tracking methods have been widely used for commercial purposes to study the best marketing strategies and profile consumer preferences, as well as in the field of home automation. In the medical field, such tools can support neuro-oculomotor investigations meant to improve the life quality of patients with neuro-motor deficits [35,36,37,38,39]. The null hypotheses for this study are that there are no significant differences between subjects of the orthodontic and non-orthodontic groups in their knowledge, expectations and perception of clear aligner therapies, and that visual attention is not influenced by the presence of clear aligners, by the characteristics of the different types of aligners or by the presence of lips. In addition, the potential clinical value of this technology, and the ethical and legal issues related to its use, have also been explored.

## 2. Materials and Methods

In a preliminary phase of the study, the researchers involved completed a month-long remote training on the technology to be used by the company that developed the smart eye-tracking app (oculid GmbH, Berlin, Germany) and followed through with a pilot test, which served as a guide to evaluate the contents to be included in the experimental test. It is worth pointing out that the study herein presented was conceived as multidisciplinary research for which adequate orthodontic knowledge is necessary. Hence, two orthodontists (one of whom is also a researcher and PhD in innovative technologies applied to dentistry and forensic medicine) with decade-long clinical experience in public and private practice were actively involved. In addition, the study was developed in collaboration with a computer engineer (currently a computer science PhD student) who had already been part of research endeavors in orthodontics and is currently involved in the development of digital orthodontic technologies. Finally, the study was coordinated, directed and supervised by two forensic medicine professors with extensive and proven experience in forensic dentistry that also extends to orthodontic problems. The sample selection was based on age (between 15 and 70 years) with no restrictions relating to gender, ethnic profile, or social, economic or cultural status. The only excluding factor was professional activity in the dental field. Dentists, both generalists and specialists, and other dental professionals such as dental assistants, dental hygienists, dental technicians, as well as students of dentistry, dental hygiene and dental technology, were excluded in order to prevent their professional skills and preferences from potentially affecting the results of the study. Ultimately, 100 subjects between in the 15–70 age range were selected (50 males and 50 females), then divided into two main groups. The first, labeled group A, consisted of 50 patients (25 males and 25 females) who went to the referral dental center for routine check-ups or non-orthodontic treatments; none of them had ever been an orthodontic patient. The other 50 patients (25 males and 25 females) made up group B, which included patients already in orthodontic therapy, former orthodontic patients and patients who had requested an orthodontic evaluation. The sample size of 100 subjects was deemed appropriate and representative, since such an amount is larger than that used in many other eye-tracking research samples [29,30,31,32,33,34,35,36,37,38]. We established the “problem discoverability” (*p*) at 0.05 and the “problem discovery goal” (*P* (*x* ≥ 1)) at 0.95, and applied the formula:

*P* (*x* ≥ 1) = 1 − (1 − *p*)^*n*^

where *n* is the sample size, and its value equals 59. In neuroscience research, a sample size consisting of 50–100 respondents is large enough to obtain comprehensive behavioral insights during emotion measurements. A sample size of 70–100 respondents is optimal for implicit priming tests (tests based on the speed of reaction to different stimuli) [40,41].

**Figure 1:**
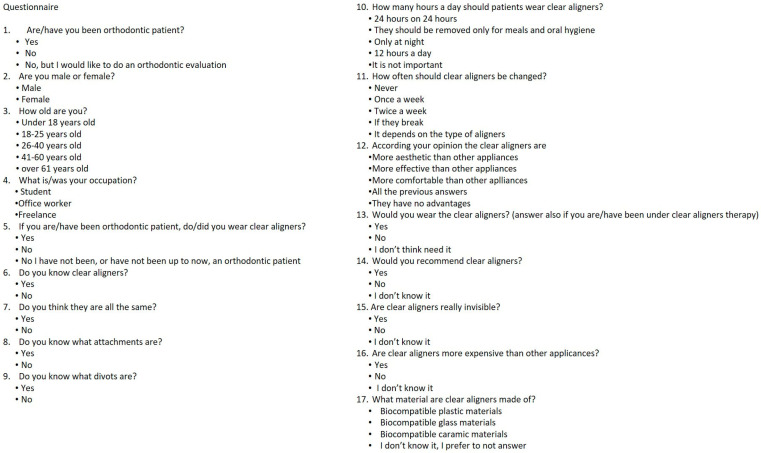
Questionnaire administered through the oculid app.

Prior to the study’s inception, an expert panel consisting of three orthodontists and three general dentists filled out the questionnaire. An expert psychometrician checked the questionnaire for common errors. Feedback obtained from experts was used to modify the questionnaire for content and validity, and a first final version was redacted. A subset of 10 participants, corresponding to 10% of the sample of subjects selected for the study, ran a pilot test of the approved version of the questionnaire. After collecting and cleaning the data, an expert statistician used principal component analysis to identify the proper number of elements the questionnaire was measuring. Then, internal consistency was reviewed using Cronbach’s alpha test. With a value of 0.90, the consistency was deemed to be adequate. Based on such criteria, the questionnaire was ultimately approved to be administered in the official test. Adult patients, or at least one parent/legal guardian of underage patients, signed a written informed consent before starting the dental/orthodontic session and/or exam. They were subsequently provided with a smartphone (Huawei (Shenzhen, China), model P smart FIG-LX1, Android system, version 9) on which the oculid app (oculid GmbH, Berlin, Germany) was installed. Through the app, participants were first given a test (in Italian and English) with a questionnaire made up of 17 multiple-choice questions, to be filled in anonymously (Figure 1). The initial questionnaire allowed for the definition of sample characteristics (sex, age range, and profession), the group to which they belonged (orthodontic or non-orthodontic), the level of their knowledge of clear aligners (characteristic components, materials, and management during therapy), and their perception of clear aligners (aesthetics, effectiveness, and costs).

**Figure 2:**
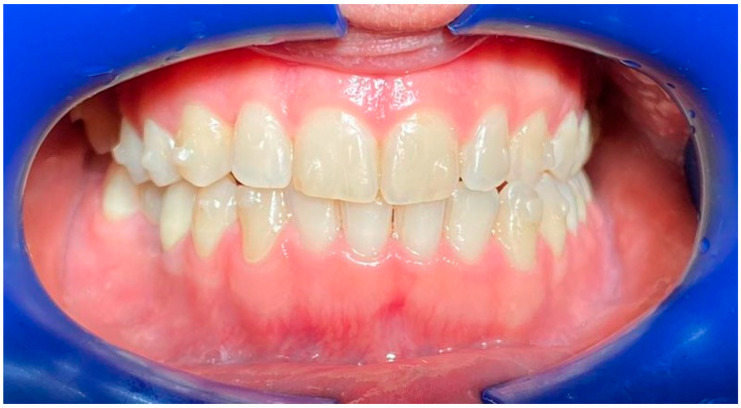
Smile 1 without clear aligners. Cheek retractors used. Anterior and posterior attachments are visible.

**Figure 3:**
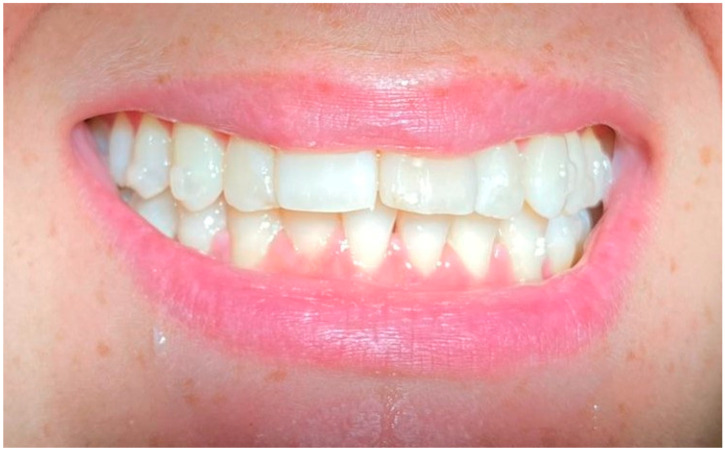
Smile 2 without clear aligners and with lips. Posterior attachments are visible.

**Figure 4:**
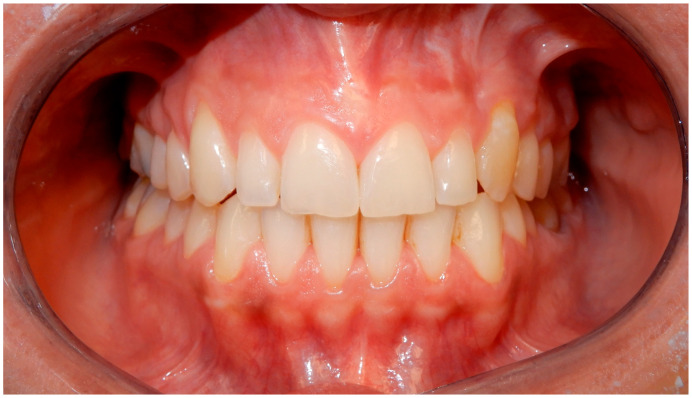
Smile 3 without clear aligners and without attachments (the system used in this case did not require them). Cheek retractors used.

**Figure 5:**
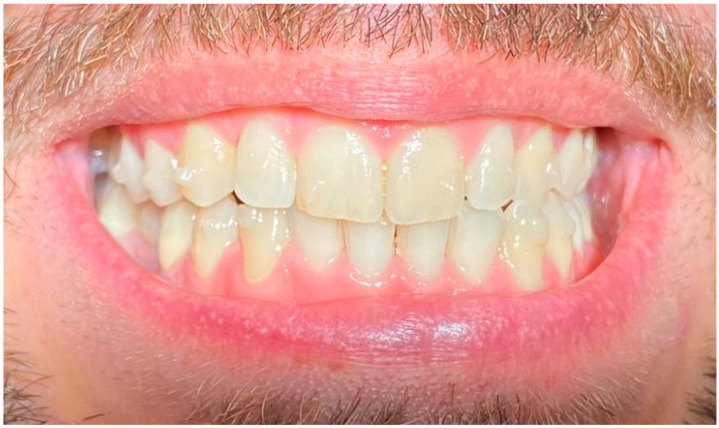
Smile 4 without clear aligners and with lips. Anterior and posterior attachments are visible (same mouth pictured in Figure 2, smile 1).

**Figure 6:**
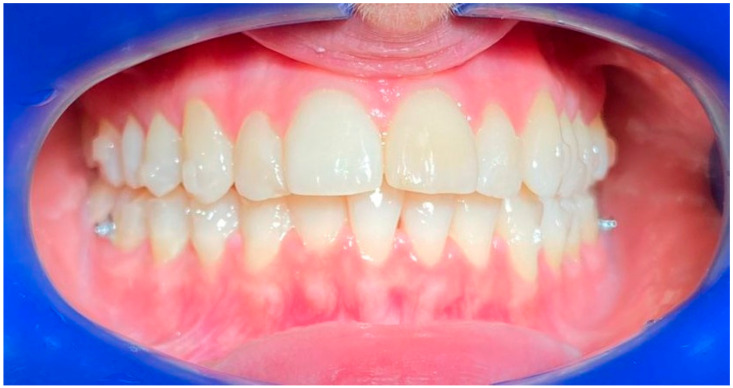
Smile 5 without clear aligners. Cheek retractors used. Posterior attachments visible (same mouth pictured in Figure 3, smile 2).

**Figure 7:**
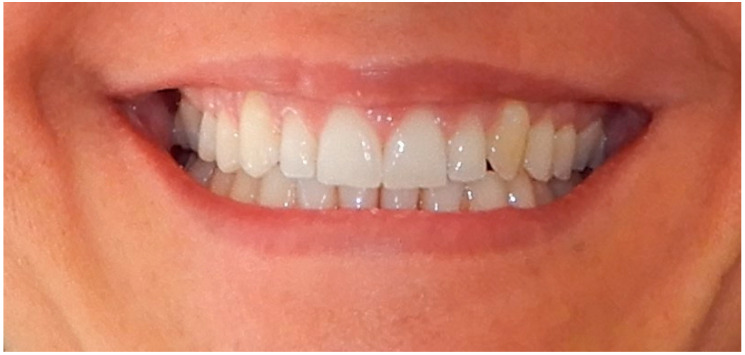
Smile 6 without clear aligners and with lips. No attachments are visible, since the system used in this case does not require them (same mouth pictured in Figure 4, smile 3).

**Figure 8:**
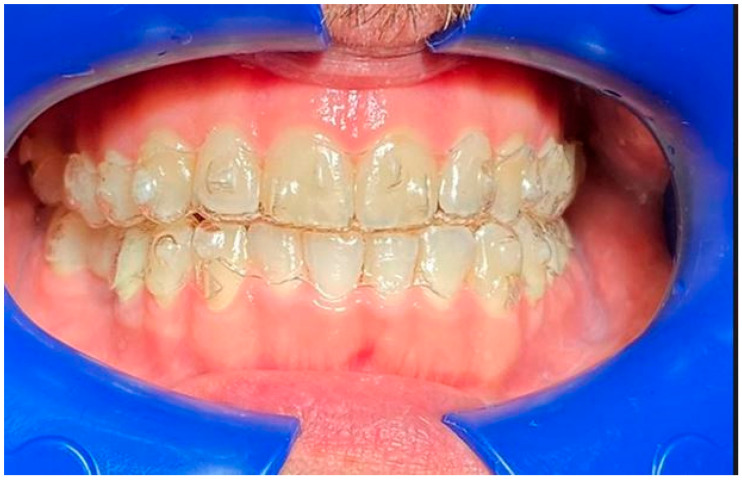
Smile 1 with cheek retractors wearing clear aligners with scalloped gingival margin and with anterior and posterior attachments (same mouth pictured in Figure 2, smile 1).

**Figure 9:**
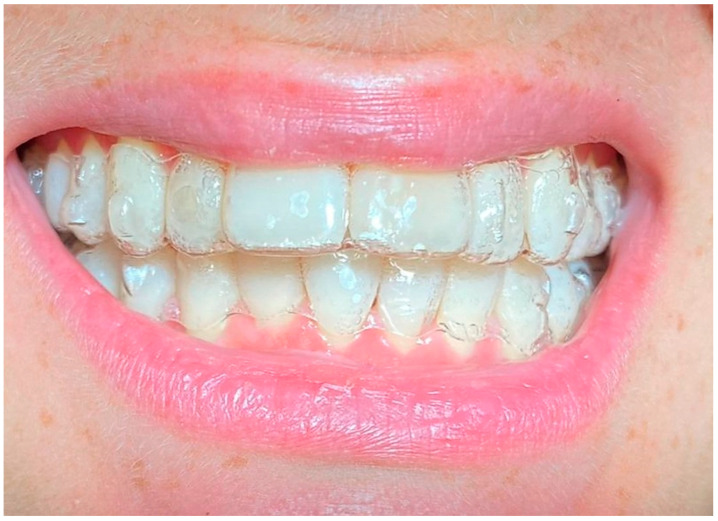
Smile 2 with natural smile wearing clear aligners with scalloped gingival margin and with posterior attachments (same mouth pictured in Figure 3 and Figure 6, smile 2).

**Figure 10:**
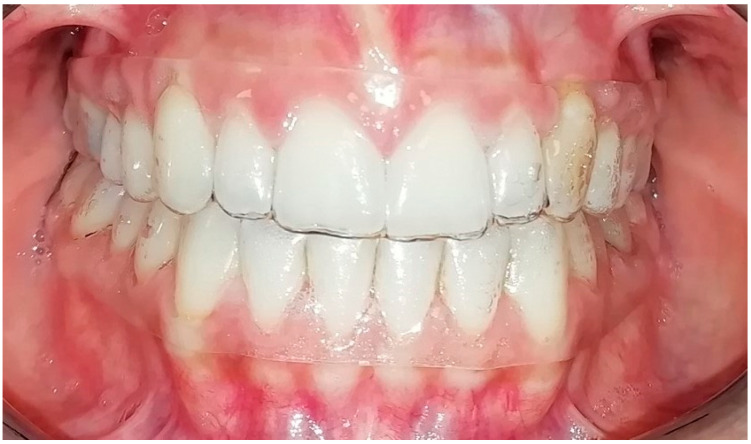
Smile 3 with cheek retractors wearing clear aligners with straight above-the-zenith gingival margin and devoid of any attachments (same mouth pictured in Figure 4 and Figure 7, smile 3).

**Figure 11:**
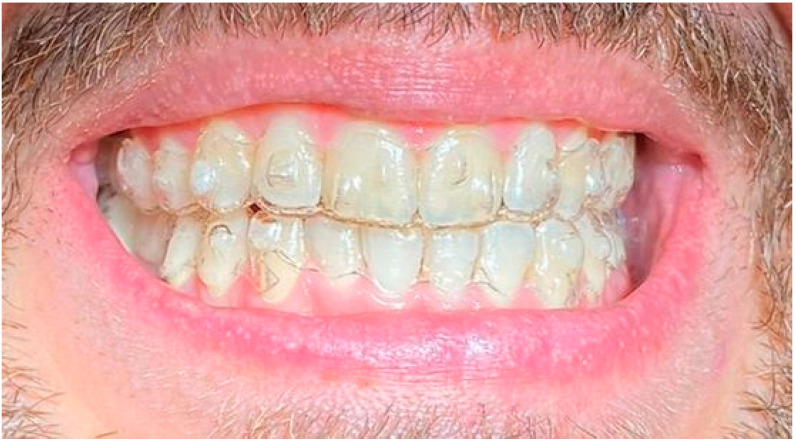
Smile 4 with natural smile wearing clear aligners with scalloped gingival margin and with anterior and posterior attachments (same mouth pictured in Figure 2, smile 1).

**Figure 12:**
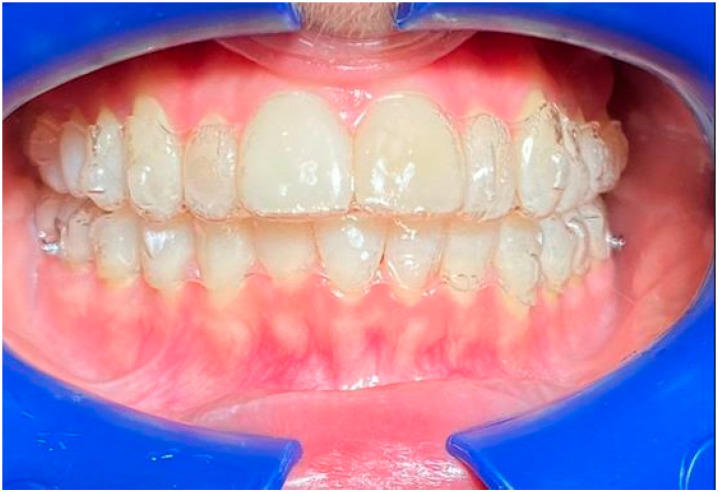
Smile 5 with cheek retractors wearing clear aligners with scalloped gingival margin and with posterior attachments (same mouth pictured in Figure 3, smile 2).

**Figure 13:**
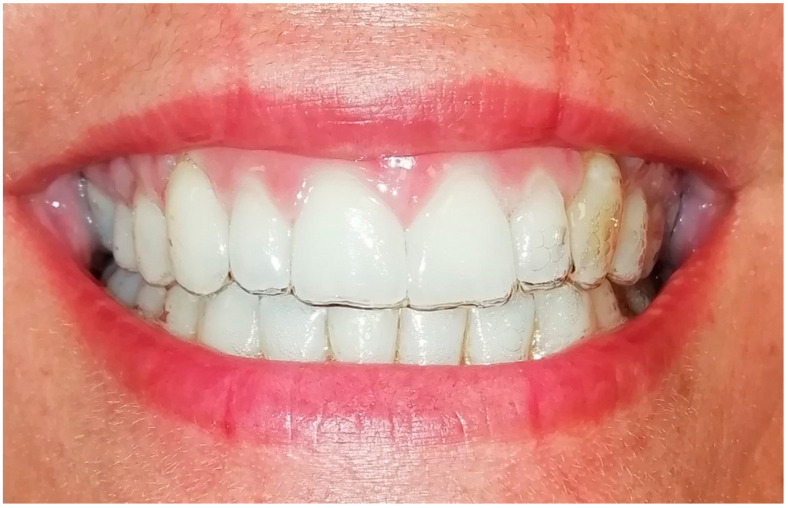
Smile 6 with natural smile wearing clear aligners with straight above-the-zenith gingival margin and devoid of any attachments (same mouth pictured in Figure 4, smile 3).

Through the same app session, respondents were asked to observe and evaluate (using star ratings from 1 to 5) 12 smiles (Figure 2, Figure 3, Figure 4, Figure 5, Figure 6, Figure 7, Figure 8, Figure 9, Figure 10, Figure 11, Figure 12 and Figure 13) with different types and designs of clear aligners (differing in terms of margin and number/distribution of attachments). The procedure that led from the multiple-choice test to the rating of the photos with smiles wearing the aligners unfolded in a guided way through explanations that the participants followed step-by-step. The process began with a calibration of eye movements, for which it was required to have adequate environmental lighting and to be without glasses. If either of such conditions went unmet, the app automatically asked for the correction of such elements, possibly also improving and maintaining the most suitable position of the face with respect to the smartphone. The average distance between the subject and the phone display was on average about 30–35 cm, as is the usual distance of use of the smartphone for other functions. The images provided in the app test belonged to three subjects whose mouths were picured with and without clear aligners (one professional photo with cheek retractors and the other with a natural smile). Two of the subjects were being treated with clear aligners equipped with attachments, only posterior in one subject and both anterior and posterior in the other, and a scalloped edge (Invisalign^®^, Align Technology, San Jose, CA, USA), and the other was in therapy with clear aligners whose system did not include attachments and was equipped with a high-margin, straight, above-the-gingival zenith (Sorridi^®^, Sorridi s.r.l., Latina, Italy). The choice of providing images with a cheek retractor or with a natural smile was consistent with the intention to evaluate the influence of the lips and their position in the evaluation and overall perception of the clear aligner based on its characteristics (design of the margin and presence and position of the attachments). The six images without aligners (with cheek retractors or with a natural smile) were used as control images during calibration and in order to assess whether the presence of aligners (in the other six experimental images) would affect the evaluation of a natural smile and one with the cheek retractor (with and without attachments). At the end of the multiple-choice test and image evaluation, respondents sent the results directly to the platform. Both the responses and the eye movements were recorded on the images with the clear aligners, which were thus processed both as partial and overall results. The identity of each participant from the platform remained untraceable, both for company operators and investigators. Before use by each patient, the surfaces of the smartphone were sanitized with a surface disinfectant spray (Detrisan AC, Mondial Snc, Limena-PD, Italy) effective against viruses (including coronavirus-type), bacteria, fungi and mycobacteria. The data collected via the multiple-choice test were organized by frequency distribution of the different response options. The data collected on the basis of the evaluation of the images were collected by a star rating for each image (1 star for null approval, 2 stars for poor approval, 3 stars for medium approval, 4 stars for high approval, and 5 stars for best approval), average fixation time of each image, average fixation time of all images, and points of interest (longest fixation time) for each image. 

In this study, a descriptive statistical analysis was carried out both for the answers to the questionnaire and for the part of the test relating to the score attributed to the images. The statistical functions in the Microsoft Office Excel software were used (Microsoft Excel 2021 version, Microsoft, Redmond, WA, USA). From the app platform, the data relating to the overall average fixation time for each image and the points of greatest interest on each image were identified as a dark red-colored thickening area (higher frequency and observation time), with the color gradient fading to orange, yellow, green, and violet, up to blue (lower frequency and time of fixation of the gaze). The chi-square test and the chi-square test with Yates correction compared the significance of the questionnaire responses relating to knowledge and opinion of clear aligners (from question number 5 to question number 16) in the two groups. The level of statistical significance was set at 0.05 (*p* < 0.05). The null hypothesis (Ho) was that there would be no relationship. To rule that out, a *p* < 0.05 (at 95% confidence) was needed. The results of the initial fixation times and longer ones by groups were compared with each other and with the overall average ones using a *t*-test (*p* < 0.05). Overall average fixation times and the average star scores for each image were compared with each other, and based on the reference group, with the following non-parametric tests: Mann–Whitney U test and Spearman’s rho test. The Wilcoxon test at *p* = 0.05 was used to compare the overall mean observation times and star scores of each image based on the evaluation of the same smile with and without cheek retractors, i.e., with the lips in a natural position. The null hypothesis asserted that the medians of the two samples or two variables compared would be identical. Several areas of interest have been identified on the images: lips (where present), front teeth, back teeth, gums and gingival margin of the clear aligner (in the photos with cheek retractors).

This research does not disclose any data which would require ethics approval. The current regulation of the ethics committee of the Higher Institute of Health [42] stipulates that projects with epidemiological, medico-social and evaluative content need assessment, approval and monitoring of trial protocols only if they contain personal data according to the legislative decrees on clinical trials and function of the ethics committees [42,43]. The official definition of “personal data” is given by the National Data Protection Authority [44], in keeping with the principles outlined in the Regulation (EU) 2016/679 of the European Parliament and of the Council of 27 April 2016 [45]. The term “personal data” includes information about first and last name, images, social security code, IP address and license plate number. The app on which the anonymous questionnaire was completed does not allow for tracing of the IP address of the person connected to the app. Moreover, the devices provided to the patients were made available by the dental care provider for each subject. Informed consent was presented on the first page of the app (and a printed version was issued to the subjects) used to perform the test. Before starting the test, each participant was asked to acknowledge that filling out the questionnaire meant that their anonymously provided answers would be used for the specific purposes of the research study herein presented.

## 3. Results

All patients completed the test correctly, and the results of all were collected from the online platform of the app, accessible only to the user registered as investigator manager soon after confirming their willingness to transmit each test to the study database.

### 3.1. Answers to the Questionnaire

**Figure 14:**
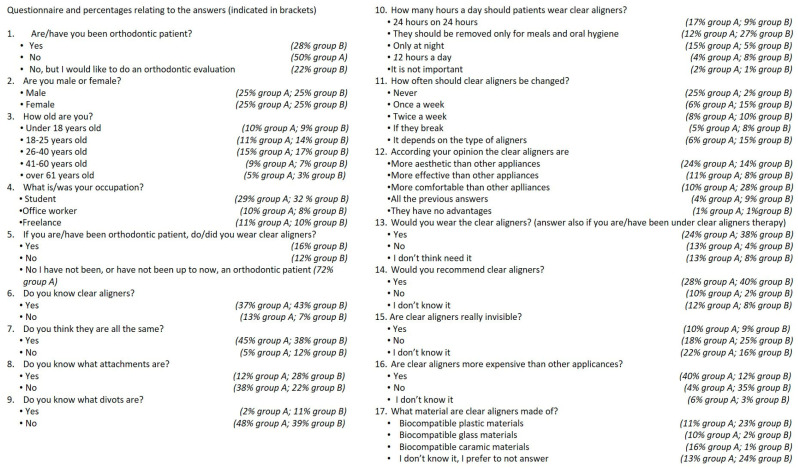
Frequency distribution of each answer in the questionnaire.

The preliminary part of the test, based on multiple-choice questions, allowed for identification among the respondents of equal shares for each sex and a similar distribution between the non-orthodontic group (group A) and orthodontic group (B); the latter group included orthodontic patients in care, former orthodontic patients, and patients who went for an orthodontic evaluation. In group B, 28% of patients were currently or previously under orthodontic treatment, and 22% of patients in such group wanted to receive an orthodontic evaluation (Figure 14). The distribution by age group in the two groups was as follows: under 18 years old, 19% (10% in group A and 9% in group B); between 18 and 25 years old, 25% (11% in group A and 14% in group B); between 26 and 40 years old, 32% (15% in group A and 17% in group B); between 41 and 60 years old, 16% (9% in group A and 7% in group B); and over 61, only 8% of patients (5% in group A and 3% in group B) (Figure 14). The population of the two groups included 29% of students in group A and 32% in group B, 10% and 8% of office workers and 10% and 11% of freelancers, respectively (Figure 8). Only 16% of patients, belonging obviously to group B, reported having worn the clear aligners during their therapy, while 12% stated they did not use them, and the remaining 72% never used them, not being orthodontic patients (Figure 14). In group A, 37% of the total study subjects responded that they were familiar with clear aligners, whereas the remaining 13% of group A subjects claimed not to be. Such a level of knowledge increased in group B, with an affirmative answer in 43% of cases versus 7% responding in the negative (Figure 14). A total of 45% of the subjects in group A and 38% of the subjects in group B agreed that clear aligners are all the same. Only 5% of patients in group A and 12% in group B believed that aligners are not equivalent in their characteristics (Figure 14). As for specific knowledge of clear aligners, only 12% of patients in group A and 28% of patients in group B stated that they knew what attachments were, versus 38% of patients in group A and 22% of patients in group B admitting that they did not (Figure 14). Knowledge of divots was found to be substantially lower in both groups. In fact, just 2% of patients in group A and 11% of patients in group B knew about divots, while 48% and 39% of both groups A and B, respectively, declared that they did not (Figure 14). A subpar level of knowledge was found regarding usage protocols for aligners. Only 12% of subjects in group A and 27% (which is the highest percentage) in group B correctly stated that clear aligners should only be removed for meals and daily oral hygiene. A total of 17% and 9% of patients in group A and group B, respectively, thought they should be worn all day, while 15% and 5% thought they should be worn while sleeping; only half a day was thought to be enough for 4% and 8%, respectively. Finally, 2% and 1% of patients in groups A and B thought the number of hours of daily use would not be an important factor (Figure 14). Regarding knowledge of the management of aligners, 25% of the subjects in group A believed that aligners should never be replaced. Such a belief was found to be significantly less common in group B, at a mere 2%. A weekly change is recognized as valid for 6% of group A and 15% of group B subjects. A bi-weekly change is considered correct for 8% of the subjects of group A and 10% of the subjects of group B. More concretely, 6% of subjects in group A and 15% of subjects in group B believed the frequency of replacement depends on the type of aligner. Finally, 5% of the subjects in group A and 8% of those in group B thought that the aligners are only to be replaced if they break (Figure 14). The clear aligners were deemed more aesthetically effective by 24% of the subjects in group A and by 14% of the subjects in group B, while they are deemed more effective for 11% and 8% of both groups, respectively, and more comfortable for 10% and 18% of the subjects of the two groups. A lower percentage, 4% of group A and 9% of group B, believed aligners to have all such advantages. On the other hand, 1% of both groups believed that aligners have no advantages (Figure 14). When patients were asked in the questionnaire whether or not they would wear clear aligners, 24% and 38% of groups A and B, respectively, responded affirmatively. In the question, it was clarified that patients in group B had already undergone treatment with aligners, or were doing so at the time of the test. Only small percentages of 13% and 4%, respectively, responded negatively, while 13% of patients in group A and 8% of patients in group B said they did not know (Figure 14). Most patients in group A and group B, 28% and 40%, respectively, said that they would recommend the use of clear aligners. In groups A and B, 10% and 2% of patients would not recommend them, while 12% and 8% were unable to answer such a question (Figure 14). Clear aligners were perceived as truly invisible devices only by relatively few subjects of the two groups: 10% of group A and 9% of group B. According to the opinion of a larger share of patients, 18% of group A and 25% of group B, the aligners are not really transparent. A rather large share of patients from both groups, 22% of group A and 16% of group B, were unable to answer this question (Figure 14). Most non-orthodontic patients, i.e., 40% of group A subjects, believed clear aligners to be more expensive than other orthodontic appliances, unlike 12% of group B patients (i.e., the orthodontic group). Conversely, most orthodontic subjects in group B believed transparent aligners to be no more expensive than other devices (35%). Only 4% of the subjects in group A did not believe them to be more expensive, while 6% expressed no opinion on that. Even 3% of the subjects within group B were unable to answer (Figure 14). Patients in group B were found to be better informed about the type of materials aligners are made of: 23% of them responded that transparent aligners are made of biocompatible plastic materials as opposed to only 11% in group A who answered correctly. A total of 10% of subjects in group A thought the aligners are made of glassy materials, while 16% of the same group believed them to be made of compatible bioceramic materials. Only 2% and 1% of the subjects of group B gave the same answers, respectively. A small percentage of subjects in groups A and B, 13% and 24%, respectively, did not know or preferred not to answer (Figure 14). 

### 3.2. Statistical Analysis Applied to the Questionnaire Answers

The chi-square test, and chi-square test with correction of Yates, whenever necessary, of independence were significant in many comparisons in order to examine the relationship between the sample group that patients belong to (non-orthodontic patients vs. orthodontic patients, including former orthodontic patients and patients interested in orthodontic therapy) and their knowledge or opinions about clear aligners (Table 1). Significantly more patients in group B, compared to group A, knew about the components of invisible aligners, such as attachments (χ^2^ = 10.16667, *p* = 0.001091 χ^2^ with Yates correction = 9.375, *p* = 0.0022) and divots (χ^2^=7.1618 *p* = 0.00747; χ^2^ with Yates correction = 5.6587, *p* = 0.017369). Patients in group B were also found to be better informed about how to use the aligners daily (χ^2^ = 14.8974, *p* = 0.004919) and how often they should be replaced (χ^2^ = 28.2214, *p* = 0.000011), and a significant statistical correlation existed regarding their positive perception of transparent aligners compared to patients of group A. In fact, patients of group B, which included those who already wore them or had used them, and also those who have already undergone therapy or would like to be evaluated to start it, would wear them (χ^2^ = 9.1165, *p* = 0.010481) and recommend them to others (χ^2^ = 8.251, *p* = 0.016156). Patients in group A believed therapy with clear aligners to be more expensive than other orthodontic therapies, with a significant difference compared to the response by patients in group B (χ^2^ = 40.7179, *p* ≤ 0.00001). Compared to patients in group A, patients in group B were also significantly more informed about the materials that make up the invisible aligners (χ^2^ = 26.0742, *p* ≤ 0.00001). Some comparisons were not significant. Although, as reported so far, the patients in group B turned out to be substantially more competent and well-informed than those in group A, the direct question on knowledge of aligners did not generate significantly different answers in the two groups (χ^2^ = 2.25, *p* = 0.133614; χ^2^ with Yates correction = 1.5625, *p* = 0.2113), nor did the question about the existence of a general, substantial difference between aligners (χ^2^ = 3.4727, *p* = 0.062389; χ^2^ with Yates correction = 2.5514, *p* = 0.110198). Even opinions regarding their greater aesthetic value, effectiveness or comfort did not show significant differences between the two groups (χ^2^ = 7.3141, *p* = 0.120194), nor did the level of the perception of real invisibility of the aligners based on the group to which the respondents belonged (χ^2^ = 2.1395, *p* = 0.343088). All the χ^2^ test and χ^2^ test with Yates correction results have been summarized in a table (Table 1).

### 3.3. Statistical Analysis Applied to First Point Gaze Time, Longest Fixation Time and Overall Gaze Time

For each image, the first point on which the gaze was fixed, the one on which it rested longer (average fixation time) and the overall observation/gaze time for each image were identified from the platform for both groups (Table 2 and Table 3). The result of the *t*-test that compared significant mean differences for the first fixation point between the two groups is not significant for both subgroups of images (without and with clear aligners worn by the subjects). The values of t are −0.37731 (*p* = 0.356915) and 1.567863 (*p* = 0.1777), respectively, for the images without and with clear aligners. The result is not significant at *p* < 0.05. The comparison between the longest gaze in the two groups is also not significant at *p* < 0.05 for both subgroups of images. The values of t are 1.27362 (*p* = 0.115803) and −1.317402 (*p* = 0.24484), respectively, for the images without and with clear aligners. The comparison between the mean values of the overall fixation time is not significant for both subgroups of images. The values of t are, respectively, 0.08133 (*p* = 0.468391) and −1.438779 (*p* = 0.20974). The comparison between the first-point gaze and the longest gaze in the A group for each smile examined at *p* < 0.05 is significant for both subgroups of images. The values of t are, respectively, −11.54622 (*p* ≤ 0.00001) and 13.291117 (*p* = 0.00004). The same comparison in the B group shows significant values (at *p* > 0.05) for both the subgroups of images. The values of t are −14.6492 (*p* ≤ 0.00001) and 13.453603 (*p* = 0.00004). Two significant results are those obtained using a *t*-test at *p* < 0.05, which compared the first-point gaze mean values for each image in both subgroups of images in the A and B group of respondents with their overall mean observation time. In the A group, the value of t is −22.47117 and the value of *p* is <0.00001 for images with patients without clear aligners; the value of t is 11.708622 and the value of *p* is 0.00008 for images of patients wearing clear aligners. In the B group, the value of t is −14.6492 and the value of *p* is <0.0001 for images with patients without clear aligners; the value of t is 17.995506 and the value of *p* is <0.00001 for images of patients wearing clear aligners. The comparison between the longest gaze during the test in group A and the overall mean observation time for each image in this group is statistically significant at *p* < 0.05 for both subgroups of images. The value of t is −20.57699 (*p* ≤ 0.00001) for the subgroup of images without clear aligners and 10.536119 (*p* = 0.0013) for the subgroup of images wearing clear aligners. The same comparison in the B group shows a value of t equal to −13.67807 (*p* = < 0.0001) for images without clear aligners and 16.270141 (*p* = 0.00002) for images wearing clear aligners, so these results are also significant at *p* < 0.05 (Table 4 and Table 5).

To evaluate whether there are significant differences in the fixation times of the two groups of subjects (group A and group B) with respect to the type of images observed (i.e., the subgroups of images with and without aligners), the *t*-test was also applied. The resulting *t*-tests (*p* < 0.05) were all significant (first-point gaze in A group: t -value is −2.84503, *p* is 0.008699; first-point gaze in B group: t-value is −1.96373, *p* is 0.038976; longest gaze in B group: t-value is −2.07183, *p* is 0.032542; overall observation time in A group: t-value is −1.99944, *p* is 0.036728), with the exception of the comparison between the longest gaze for the two series of images in group A (t-value is −0.90427; *p* is 0.193559) and the overall observation time in group B (t-value is −1.74829; *p* is 0.055493) (Table 6).

### 3.4. Statistical Analysis Applied to Star Rating, First-Point Gaze Time, Longest Fixation Time, Overall Gaze Time

After viewing each image with no time limit, the subjects involved in the study expressed a star rating (Table 7 and Table 8). Based on the average scores assigned, it is possible to see that smile number 1, the one in which anterior and posterior attachments are seen in the presence of cheek retractors, is the one with the lowest scores from the two groups when clear aligners are worn. The star rating for smile number 2 (in which the lips are visible) is similar whether the aligners are worn or not, and only the anterior attachments are visible. Smile 3, which matches smile 6 with lips in natural expression, and smile number 6 have the highest average scores, but the star rating improves when clear aligners are worn. Smile number 5, in which attachments are always present anteriorly, has a similar star rating whether the clear aligners are worn or not. Smile 4, equal to smile 1 but with lips, has a better star rating in the photograph without clear aligners. The Mann–Whitney U test was used to assess the significance of differences in star ratings between the groups, and between the groups based on the subgroup of images. The U value ultimately ranged from 14 to 18, with the critical value of *p* < 0.05 at 5. Therefore, the result was not significant at *p* < 0.05. Such findings indicate that the evaluation of the images is not significantly different in the two groups and that the presence or absence of aligners (both in the intraoral vision with the cheek retractors and in the image with lips) does not influence the overall average judgment. Spearman’s rho is a non-parametric test used to measure the strength of the association between two variables, where the value r = 1 means a perfect positive correlation and the value r = −1 means a perfect negative correlation. This test was used to measure the strength between the mean rating in stars for each image (both without and with clear aligners worn) for the two groups with first-point gaze mean values, longest gaze means values and overall observation time mean values. The correlation at *p* < 0.05 is not significant in both the groups for all images when the mean star value ratings are compared to first-point gaze mean values (for images without clear aligners worn in group A, rs is −0.2354 and *p* is 0.65343, while in group B, rs is −0.54286 and *p* is 0.2657; for images with clear aligners worn in group A, rs is −0.63754 and *p* is 0.17326, while in group B, rs is −0.48571 and *p* is 0.32872) and to longest gaze mean values (for images without clear aligners worn in group A, rs is −0.02942 and *p* is 0.95588, while in group B, rs is 0.25714 and *p* is 0.62279; for images with clear aligners worn in group A, rs is 0.5161 and *p* is 0.29458; in group B, rs is 0.2 and *p* is 0.704). The strength of the correlation becomes significant in the comparison when mean star value ratings are compared to the overall observation time in both groups when clear aligners are worn (in group A, rs is −0.94112 and *p* is 0.0051; in group B, rs is −0.088571 and *p* is 0.01885), suggesting that the overall observation time influences the final rating in stars (Table 9), while the overall observation time and star rating do not influence each other in the assessment of images without clear aligners (for images without clear aligners worn in group A, rs is −0.47079 and *p* is 0.34599, while in group B, rs is −0.77143 and *p* is 0.0724). The Wilcoxon test (*p* < 0.05) was used to assess whether the overall mean observation time and the mean star score are influenced by the presence of lips or rather by a professional intraoral vision with cheek retractors, and by the presence or absence of clear aligners in the same smiles without and with cheek retractors. Both evaluations are statistically significant for images with clear aligners worn (Table 10). In the first case comparing the presence of lips or cheek retractors with the overall observation time, the value of z is −1,9917 and the value of W is 1. The critical value for W at N = 6 (*p* < 0.05) is 2. The overall observation time is significantly higher in the images with cheek retractors. The presence of lips led to significantly shorter observation time. In the second case, the presence of lips significantly influences the evaluation in stars, and scores are higher than in the same smiles with cheek retractors. The value of z is −2.0026. The value of W is 0. The critical value for W at N = 5 (*p* < 0.05) is 0 (Table 10). Results are not significant when the Wilcoxon test is applied to the overall mean observation time (W-value is 10 and z-value is −0.1048) and mean star score of the images (W-value is 6 and z-value is 0.9435) without clear aligners in both groups of respondents. These results point to clear aligners as a significant and different influence on overall fixation time and star rating, depending on whether the mouths with transparent aligners are more exposed to visual attention due to the presence of cheek retractors or are perceived as more natural due to the visible lips. Observation times are, therefore, significantly associated with the presence of aligners (W is equal to 7 and the z-value is −2.5103); in particular, such time spans increase significantly in the presence of cheek retractors (the value of W is equal to 1 while the z-value is −1.9917). The observation time does not significantly change in the presence or absence of aligners when the smile is framed by lips (W is equal to 4 and the z-value is −1.3628). The star score is not influenced by the presence of aligners (W is equal to 27.5 and the z-value is 0), nor is a relationship found with the use of cheek retractors (W has a value of 5.5 and the z-value is −0.5934) or with observation in the presence of lips (W is equal to 5 and the z-value is −0.6742) (Table 10).

### 3.5. Statistical Analysis Applied to Initial Fixation Points and Longer Fixation Points in the Different Areas of Interest for Each Image

**Figure 15:**
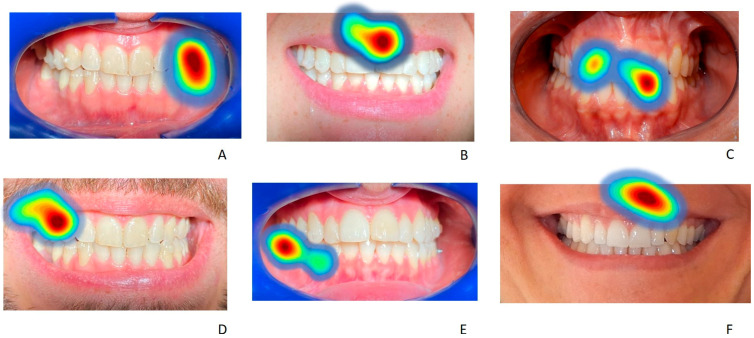
Examples of heatmap screenshots of some of the most frequent average fixation points in the different areas of interest for each image examined of subjects without clear aligners. In (**A**,**D**,**E**) the average fixation point is located in the lateral area with posterior attachments, in (**B**,**F**) it is located in the upper lip, in (**C**) it is located in the front area without attachments.

**Figure 16:**
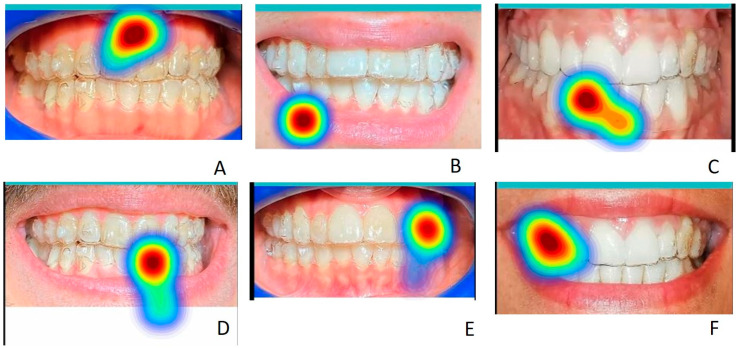
Examples of heatmap screenshots of some of the most frequent average fixation points in the different areas of interest for each image examined (from the first to the sixth smile) of subjects with clear aligners on. In (**A**) the average fixation point is located in the front area with anterior attachments in the upper teeth; in (**B**) it is located in the lower lip; in (**C**) it is located in the front area corresponding to lower teeth without attachments; in (**D**) it is located in the front area corresponding to lower teeth with attachments; in (**E**) it is located in the lateral area with attachments, in (**F**) is located in the lateral area without attachments.

The initial average fixation points and those of longer duration for each image, in the different areas of interest, are displayed as a heat map with a color gradient ranging from red to light blue (Figure 15 and Figure 16). In some cases, the observed images have a significantly different frequency distribution using the chi-square test (*p* < 0.05), both compared to groups A and B and compared to the observation of images of smiles without aligners and with aligners worn.

### 3.6. First Gaze Point and Areas of Interest: Comparisons Based on Groups and Images

The presence of attachments, anterior and posterior or only posterior, affects the position of the first gaze point in both groups A and B, both in the images with aligners and in those without aligners and the presence of the lips rather than cheek retractors is a distraction from the attention paid to these elements of the aligners (Table 11). In both groups, for the image of smile number 1 (with and without cheek retractors and anterior and posterior attachments, and with a scalloped gingival margin), the most frequent first observed point is in the area of the anterior attachments in 63% of cases when clear aligners are worn and in 57% of cases when clear aligners are not worn (Table 11). The χ^2^ test is not significant for the comparison of the first-point gaze related smile 1 between the A and B groups (Table 12 and Table 13), but is indeed meaningful for the comparison between the images without and with clear aligners (χ^2^=17.5352; *p*-value = 0.040965) (Table 14). Smile 2 has a higher percentage of the first gaze point on the lower lip (52%) when clear aligners are worn for group A and on the lower lip when clear aligners are not worn for group B (30%). The χ^2^ test is significant in the comparison between groups when clear aligners are worn (χ^2^ = 19.8667; *p*-value = 0.000531), and when we compare the first-point gaze of smile 2 in images with and without clear aligners (χ^2^ = 26.348; *p*-value = 0.00958) (Table 12 and Table 13). For the third smile, the most frequent gaze points are the anterior teeth without clear aligners for group A (45%) and the gingival margin without clear aligners worn for group B. The χ^2^ test is significant only in the comparison between images without and with clear aligners (χ^2^ = 13.2495; *p* = 0.03924) but not between the A and B groups. In the images with the fourth smile, the first gaze point is more frequent in the anterior attachments when clear aligners are not worn for group A (37%) and when clear aligners are worn for group B (33%) (Table 11). The χ^2^ tests are positive both in the comparisons between groups (χ^2^ =10.839, *p*-value = 0.05466725; χ^2^ = 4.085, *p*-value = 0.53724414) than in the comparison between images of smile 4 without and with clear aligners worn (χ^2^ = 16.991; *p*-value = 0.31940271) (Table 12, Table 13 and Table 14). In the fifth image, the most frequent first gaze point for group A is on the posterior attachments when clear aligners are not worn (50%). In the group B, the anterior teeth without attachments follow in order of frequency (41%), as with the first gaze point in the image without clear aligners (Table 11). The χ^2^ tests are positive in their comparisons between groups (χ^2^ = 7.5256, *p*-value = 0.023218; χ^2^ = 9.7966, *p*-value = 0.007459) (Table 12 and Table 13). In the sixth smile, the most frequent first-gaze spots are located on the upper lip (image with clear aligners worn) for 32% of group A subjects and on the lower lip (image of smile 6 without clear aligners worn) for 31% of group B subjects (Table 11). The χ^2^ tests in this case are all not significant (Table 12, Table 13 and Table 14). 

### 3.7. Longest Gaze Point and Areas of Interest: Comparisons between Groups and Images

The statistical evaluation of the longest gaze for areas of interest was conducted in the same way as the first gaze point. Smile 1 has a higher percentage on the anterior attachments in both groups (56% for group A and 58% for group B) without and with clear aligners (Table 11). The χ^2^ tests in this case are all not significant (Table 12, Table 13 and Table 14). In the second image, 36% of the subjects in both groups fix their gaze longer on the upper lip with and without clear aligners worn. The χ^2^ tests in this case are all not significant (Table 12, Table 13 and Table 14). In the third image, 46% of the subjects in group A and 42% of subjects in group B observe the gingival margin longer. In the fourth image, 30% of the subjects in group A fix their gaze longer on the anterior attachments in the image without clear aligners, and 37% of the subjects in group B fix their gaze longer in the fourth smile with clear aligners placed on the posterior attachments (Table 11). The χ^2^ tests in this case are significant (Table 12, Table 13 and Table 14) both in the comparisons between groups (χ^2^ = 2.801, *p*-value = 0.73063284; χ^2^ = 13.093, *p*-value = 0.02252267) and in the comparison between images of smile 4 without and with clear aligners worn (χ^2^ = 26.08, *p*-value = 0.03719016) (Table 12, Table 13 and Table 14). In the fifth image, 64% of subjects in group A fix their gaze longer on the posterior attachments in the image where clear aligners are worn, while 45% of subjects in group B fix their gaze longer on the anterior teeth without attachments when clear aligners are not worn (Table 11). The χ^2^ tests in this case are significant (Table 12, Table 13 and Table 14) both in the comparisons between groups (χ^2^ =25.3514, *p*-value < 0.00001; χ^2^ = 20.5001, *p*-value = 0.000035) and in the comparison between images of smile 5 without and with clear aligners worn (χ^2^ = 46.4368, *p*-value < 0.00001) (Table 12, Table 13 and Table 14). In the sixth image, 42% of subjects in group A fixed their gaze longer on the upper lip in the image without clear aligners worn, while 31% of the subjects in group B gazed longer at anterior teeth with clear aligners on (Table 11). The χ^2^ tests in this case are significant (Table 12, Table 13 and Table 14), both in the comparisons between groups (χ^2^ = 19.6296, *p*-value = 0.000591; χ^2^ = 27.2657, *p*-value = 0.000018) and in the comparison between images of smile 6 without and with clear aligners worn (χ^2^ = 48.4195, *p*-value < 0.00001) (Table 12, Table 13 and Table 14). 

A post-hoc comparison for multiple tests was carried out by first calculating the one-way ANOVA and, subsequently, applying the Tukey HSD, Scheffé, Bonferroni and Holm multiple comparison tests. The *p*-value corresponding to the F-statistic of the one-way ANOVA applied to the comparison between the first point of gaze and the longest gaze in the images without and with clear aligners worn is lower than 0.05 in both of the analyzes (respectively, the *p*-value is 0.0018 and 0.0039 in the two different ANOVAs); indeed, the two comparisons are significantly different in both evaluations (Table 15 and Table 16). All the post-hoc tests for multiple comparisons applied found a significant difference in the first-point gaze-based comparison between smiles 3 and 4 and between smiles 4 and 5 (Table 17). In the longest gaze-based comparison, all the post-hoc tests for multiple comparisons applied found a significant difference between smiles 3 and 4 and between smiles 4 and 5 except in the Scheffé T-statistic (Table 18). 

## 4. Discussion

Clear aligners have undoubtedly marked a major turning point in orthodontic therapeutics. Many patients who seek to improve their smiles have forgone multibracket orthodontic treatment, deemed to be aesthetically lacking [4,7,22,23]. The alternative using ceramic or composite resin aesthetic brackets was well received, but certainly the most welcome option is removable and transparent aligners [26,29,30,31]. Clear aligners are now widely used to treat not only simple cases, but also moderately complex or severely complex cases, even in the form of hybrid therapies [46,47,48,49]. In fact, clear aligner systems have substantially evolved both from a mechanical standpoint, with respect to the strategies that can be implemented to move teeth, and from a design and manufacturing perspective. Various companies all over the world have, in fact, developed exclusive systems and protocols to differentiate themselves from their competitors [50,51]. With a few exceptions, most count among the main strategies the application of resin buttons, mostly called “attachments”, which have different shapes, distributions and dimensions according to their indications [52]. Some systems have instead aimed to reduce or even eliminate attachments altogether, opting for less visible elements which some studies have shown to be better performing, e.g., “divots” [52,53,54]. These are small punctiform introflections in the thickness of the aligner which, inserted using different configurations, can effectively guide most of the movements guided by traditional attachments. It is also necessary to recognize that the application of attachments can entail operator–employee errors, potentially jeopardizing favorable therapeutic outcomes. This occurs above all due to inadequate application protocols, which cause early detachment, a defect in the resinous material or, even more commonly, an excess of resinous material which would increase the areas of discontinuity in the tooth–aligner interface, compromising the effectiveness of the treatment [51,55]. The design of the gingival margin of the aligner can make it better or worse, and it may be no coincidence that the current trend is to use aligners with straight margins rather than scalloped [50,53,54,56]. Some studies have also analyzed the colorimetric changes suffered by aligners during repeated contact with commonly used foods or drinks that have the ability to stain teeth and aligners. This can have an impact on both the stability of the material and its aesthetic perception [57,58].

This study goes beyond assessing the ability of the different systems to carry out more or less effective treatments according to their constitutive characteristics; rather, it is designed to focus on a method of evaluating the aesthetic perception of the clear aligner based on its look. Such an evaluation is not merely based on collecting answers to a set of questions, but rather relies on neuroscience. Smart eye-tracking technology makes it possible to objectify and quantify the attention of a subject’s gaze upon receiving certain visual stimuli [29,30,31,32,33]. While its use in the medical and dental fields is not new, the way in which such techniques were utilized in this study is new to date. Studies centered around this type of gaze assessment place the subjects involved in front of a PC with a webcam or special glasses capable of recording the movement of their gaze [29,30,31,32]. There are discussions in the literature on the possible weaknesses of the results thus obtained, due to the fact that stress, discomfort, and awareness of being in a controlled environment can alter the quality of visual attention [33,34,35,36,37,38]. Smartphones, on the other hand, act as catalysts for visual attention, making the subjects feel at ease, as if external interference were not perceivable, but rather as if an immediate and engrossing relationship between subject and device were more obvious [59,60,61]. Recent studies on the perception of orthodontic therapies have involved both patients and dentists or orthodontists, emphasizing the fundamental importance of comfort and quality of life for the former, while for clinicians, emphasizing the greater importance of clinical performance and the results obtained [9,29,30,62]. In our study, we decided to include only non-orthodontic and orthodontic patients and to exclude dentists and specialists precisely because asking clinicians to aesthetically evaluate aligners might not be meaningful, since personal knowledge and preferences can understandably influence both the answers to a questionnaire and gaze analysis. Other studies have documented the meaningful differences that exist between orthodontists and general dentists in judging perspectives and perceptions of therapies using clear aligners and in assessing the complexity of cases on which the therapeutic choice then depends [63,64,65]. Surely, the clear aligners are perceived by patients more positively than fixed multibracket therapy with respect to the treatment process [66], and that holds true based on our study where both groups would wear aligners, consider them better overall than other systems, and would recommend them to others, although for the non-orthodontic group, clear aligners are perceived to cost more than other types of braces. In our study, most of the subjects in both groups answered that they knew about clear aligners, but the vast majority believed that there were no differences between the various systems. Although the orthodontic group was more informed about attachments, in both groups different levels of knowledge were found for divots. Such specific knowledge, like that of materials, has not been evaluated in other studies [26,29,30], and neither have other kinds of specific knowledge on aligner handling and maintenance, on which patients in the orthodontic group were more conversant. The distribution by type of occupation was fairly uniform in the two groups, but there was certainly a greater presence of young subjects, i.e., still students. Such a finding is noteworthy when evaluating the results of the study because even if the age distribution is higher among subjects aged between 18 and 40, this would mean that a significant share of them are either unemployed or still in education/training. The social–environmental condition, much like age, can influence opinions regarding orthodontic therapies with aligners or other devices. Various studies have in fact found adults or young adults to prefer therapies with transparent aligners, as opposed to adolescents and children who tend to prefer other types of devices [22,23,24]. From a technical standpoint, smart eye-tracking technology strongly relies on on gaze fixation times. Other studies have highlighted the importance of the first fixation point as a comparative element of visual attention between subjects and different visual stimuli [30,31]. In our study, no significant differences were found in the two groups between the average times of the first observation point, the longest one and of the overall observation of each image. This could indicate that the average observation time on the first point detected is so short as to not influence the longer fixation times and those on each image overall, thus reducing the importance of the role of the first point of gaze when assessing visual attention of the subject, which is instead stressed by another study [31]. Such results are confirmed when we combine the objectivity of the gaze detected by the technology used in this study with the subjective expression of liking the image expressed as a score rated in stars. In both groups, such a score is closely related to the overall fixation time of the image rather than to the first point of gaze or to the longest one, indicating that the subjects construct their own aesthetic opinion on a given image probably not from the first observation point or from the element that they have observed the longest, but rather from the image which they are observing as a whole. Such a conclusion, however, seems to conflict with the results of other studies which, using smart eye-tracking technology in dentistry, state that the first point of gaze coincides with the preferences expressed in the questionnaires [29,67]. Another aspect unveiled by our study has to do with the presence of lips, which significantly reduces the overall observation time of smiles with aligners, which time increases equally significantly when the observers are offered a view of the same smiles with cheek retractors, as is the case with professional intraoral photos. Such a finding is meaningful when compared with other studies which, while not making use of this oculometric technology, underline the influence of the lips in the perception of the attractiveness of a smile [1,10,13,21]. In a study on the perception of aesthetic appliances, including ceramic brackets and aligners with and without attachments, aligners with attachments were found to be observed longer together with ceramic brackets than those without attachments, and generally longer both in full-face photos and smile close-ups [30]. Our study was designed to adapt the principle of the areas of interest of such smile close-ups [30] to the characteristics of the smiles which we took into account. In fact, using the cheek retractors, we also considered the gingival area to which the gingival margin of the aligner should correspond in different heights (depending on the type of aligner). We also distinguished upper and lower lip, anterior and posterior attachments and anterior and posterior teeth intended as teeth covered by aligners without attachments. We have not taken into account the differences in the buccal corridors of the various photos presented, although they can influence the aesthetic perception of the face and smile [11], because the heatmaps never revealed areas corresponding to them (in red) in which the initial fixation time, or longer or overall time, was greater than in other areas. Indeed, the heatmaps at the corners of the mouth refer to teeth with and without attachments, and this is confirmed by selecting the visual pinpoint representation instead of the heatmap on the app dashboard. The gingival margin significantly drew the attention of the observers of both groups, for instance, in the photo of the smile wearing the aligners of the system without attachments; subjects in the orthodontic group had longer observation times overall. In the same image, in the presence of lips, the upper lip is observed first and longest. No studies have yet focused on the visual attention paid to such elements (lips and gingival margin), hence it can only be inferred that in this study, the presence of attachments negatively affected both aesthetic preferences and observation times regardless of the belonging group, as was the case with the smart eye-tracking technology study on the aesthetic perception of attachments [31]. The anterior attachments, where present, were observed for longer than the posterior ones, and only the presence of lips distracted the observer from their presence. Aligners without attachments achieved the best aesthetic evaluation and longest observation times. Posterior attachments, in the absence of other attachments, were observed more frequently in both groups only as the average first point of gaze, but were never the longest point observed. The most frequently observed lip is the upper lip, regardless of the type of smile and lip shape. The comparison between the observations of the same images without worn aligners allowed us to have a group of control images which showed that, in general, the presence of worn or unworn aligners, and therefore in which only the attachments (if provided) are visible, does not significantly influence the judgment of smiles. Conversely, observation in the presence of cheek retractors, with aligners on, influences the judgment more (which becomes more negative). The overall observation time in relation to the score attributed to smiles also varied significantly in the two groups for the images with aligners on; that seems to suggest that the presence of aligners increases the total observation time and affects judgement. Where present, attachments, whether in images with aligners on or off, tend to constitute the first point of gaze and the one with the longest fixation. In the presence of anterior and posterior attachments, the anterior attachments are observed longer. This holds true for the same smiles where the attachments are visible but the aligners are not worn. In such instances, the only distracting element seems to be the lips. Ultimately, the difference between our study and others currently available on smart eye-tracking technology, in the dental and non-dental fields, lies in the fact that we used a smartphone and not a dedicated workstation as the other studies have [29,30,31,32,33,34,35,36,37,38,67], in which gaze was tracked, recorded and then analyzed by devices that support this technology in a non-integrated way, but rather through special glasses, webcams and specifically developed external sensors. The real invisibility of the technology that we used for this study, as well as its usability and ease of use, give rise to ethical and legal implications. In fact, such concerns had already been raised when it became apparent that improvements in the performance of the algorithms used to determine and track eye movements would become increasingly cheap and less bound by specific hardware supports, and eye-tracking technologies would considerably spread as a result [68]. Despite the enormous benefits which such innovations will likely produce, their widespread use does entail substantial risks linked to data gathering which, through the recording of eye movements, can jeopardize the privacy of those who, even unknowingly, find themselves sharing personal, sensitive information [69]. Eye-tracking devices can in fact record a large variety of gaze parameters. In fact, not only do they record eye movements such as fixations, saccades and smooth pursuit eye movements but also other ocular activities such as the average distance between the eyelids, blink frequency and duration, pupil size and distance as well as pupil reactivity [70]. Facial features around the eyes can also be recorded (e.g., skin color, presence of wrinkles, moles, and facial expressions). All these elements, like fingerprints and palatal rugae, are unique characteristics of the subject and contribute to defining the subject for biometric identification (including age, gender, and ethnicity) [71]. This information can also be detected when the sensor is a common smartphone camera, as in this case [68]. In fact, it is no coincidence that “iris recognition” is one of the most widely used methods in surveillance and security systems on a global level (e.g., eye scans at airports) [72]. The possible violation of biometric data is not the only ethical and medico-legal implication. Through eye movements, pupil dilation and so-called “eye blinking”, it is also possible to determine the type of mental activity of a subject at that given moment (calculations, learning, memorization, problem solving, etc.), as well as stress levels and emotional state (agitation, relaxation, depression, or altered mood) [73,74,75,76]. Eye-tracking has also been used to assess the degree of human experience in performing certain tasks and understanding their meaning [77,78,79]. Such an approach has been used in medical, sport and school settings, and it is worth stressing that this analysis can have a predictive value on individual learning curves and levels of performance [80,81,82,83,84]. In our study, we use smart eye-tracking technology for one of its most common applications, i.e., to profile individuals based on what they like or dislike. While this may not pose major concerns, it is worth considering that the extensive use of such technologies can also detect our aversions and phobias, and such data may be illicitly exploited [85]. Other information can be worked out on individual psychological, mental and physical states of health, which can also be a double-edged sword [86,87]. If, on the one hand, the technology is valuable to us from a medical perspective, e.g., to evaluate visual attention features in patients suffering from numerous pathological conditions and to assess substance abuse repercussions, then on the other hand, the same information can be recorded and used to exploit the vulnerabilities of frail individuals [88,89,90,91]. The data detectable through gaze tracking, even through a common smartphone [92] (from which, among other things, the ambient background sounds and the activities extrinsic to the app itself, such as other apps simultaneously running on the device, can be extracted), led us to opt for a single smartphone dedicated to this app throughout the study. In so doing, we were able to avoid any issue possibly arising from the installation of the app on patients’ personal devices, in light of the fact that the personal information detectable by this type of technology falls within the “special category data” protected by the EU’s General Data Protection Regulation (Article 9 GDPR) [44]. It is worth stressing that the app used in this study has a regulation that is as simple as it is armored in terms of user safety, because it is structured in the remote access platform in such a way as not to allow the participants’ personal and sensitive data to be traced. The research or account manager can only view the collected data broken down according to the categorizations, as indicated and established before the start of the study, and the screen recordings of app proceedings at the time of the test, with the relative fixation points reproducible as heat maps, dots, etc. There is no way to view or download the phases relating to the calibration of each subject’s gaze. Furthermore, precisely due to the regulation that has codified the GDPR prescription, all data relating to tests carried out are canceled after four months, with no way to retrieve them. Despite the risks associated with privacy violations, the benefits of eye-tracking technology are undisputed when placed under professional supervision and for the positive applications for which it has been developed and implemented over the years [90,91]. Its use should be more thoroughly scrutinized and regulated by evidence-based, international legal frameworks and privacy enforcement bodies. In the meantime, information technology companies that offer such services and the users, including clinicians who make use of these promising and valid tools for understanding patients, should use them responsibly and put in place all possible measures to prevent sensitive information of third parties from being disclosed, even accidentally. The proliferation and constant improvement of such technologies, made even more accessible by newly-developed dedicated apps, calls for even stricter standards of safety and reliability [68,92]. This study has potential limitations. In fact, the lack of similar studies assessing such a technology on smartphones makes it currently the first one to have raised questions from our experience and to have called attention to the possibility of easily using such a complex technology even in daily activities. On the other hand, however, given the current absence of a frame of reference in the form of similar studies, further research contributions are needed involving the same mobile technology in order to shed a light on its potential as well as its flaws/limitations.

## 5. Conclusions

The use of app-based smart eye-tracking technology is a very versatile and well-accepted strategy for patients to learn more about their information and opinions relating to clear aligners. Gaze tracking, along with the answers provided during the preliminary test, enabled us to validate this tool as a communication strategy. Essential to that end was also the evaluation of the different types of smiles with aligners, which we had the subjects view and assess. As for aesthetics, our findings show that patients tend to judge aligners without attachments more positively. However, the observation of a natural smile, in which the aligners are framed by lips, has always been found to improve aesthetic perception of the aligner, regardless of the presence and position of the attachments. Orthodontic patients, or those considering orthodontic treatment, are mostly well informed on the constitutive characteristics of aligners and therapeutic management. However, aligners are also judged equally positively by patients outside of the orthodontic group. The ethical and legal issues arising from smart eye-tracking technologies are noteworthy, and should not be underestimated. Careful, responsible and competent use is the only viable way to protect both the patients who resort to such tools and healthcare operators in order to fully harness the added value of this technology, which, from a neuroscience-based perspective, has found promising applications in everyday life.

## Figures and Tables

**Table 1:** Significance of the relationship between group membership and knowledge and opinions on clear aligners.

Questions	Chi Square Test	*p*-Value	Meaning	Chi-Square Test with Yates Correction	*p*-Value	Meaning
Do you know about clear aligners?	2.25	0.133614	Not significant	1.5625	0.2113	Not significant
Do you think they are all the same?	3.4727	0.062389	Not significant	2.5514	0.110198	Not significant
Do you know about attachments?	10.6667	0.001091	Significant	9.375	0.0022	Significant
Do you know about divots?	7.1618	0.007447	Significant	5.6587	0.017369	Significant
How many hours a day should patients wear aligners?	14.8974	0.004919	Significant	Not Applicable	0	0
How often should aligners be changed?	28.2214	0.000011	Significant	Not Applicable	0	0
How would you define clear aligners?	7.3141	0.120194	Not significant	Not Applicable	0	0
Would you wear clear aligners? (answer also if you are under clear aligner therapy)	9.1165	0.010481	Significant	Not Applicable	0	0
Would you recommend clear aligners?	8.251	0.016156	Significant	Not Applicable	0	0
Are clear aligners really invisible?	2.1395	0.343088	Not significant	Not Applicable	0	0
Are clear aligners more expensive than other appliances?	40.7179	<0.00001	Significant	Not Applicable	0	0
What material are clear aligners made of?	26.0742	<0.00001	Significant	Not Applicable	0	0

**Table 2:** Fixation time in seconds (mean values in seconds) related to images without clear aligners worn.

	First-Point Gaze		Longest Gaze during the Test		Overall Observation Time	
Smile	Group A	Group B	Group A	Group B	Group A	Group B
1	0.16	0.28	1.30	1.35	24	22.7
2	0.17	0.12	1.87	1.71	19	21
3	0.13	0.19	2	1.50	21	16
4	0.18	0.13	1.23	1.25	17.9	16
5	0.12	0.10	1.67	1.44	21.3	22.6
6	0.10	0.11	1.86	1.56	19	23.1

**Table 3:** Fixation times in seconds (mean values in seconds) related to images with clear aligners worn.

	First-Point Gaze		Longest Gaze during the Test		Overall Observation Time	
Smile	Group A	Group B	Group A	Group B	Group A	Group B
1	0.26	0.39	1.43	1.36	33	27.2
2	0.23	0.22	2.1	1.82	25	23
3	0.19	0.25	2.13	1.91	20	19.6
4	0.24	0.26	1.96	1.72	27	25.5
5	0.14	0.17	1.51	1.83	26.3	26
6	0.18	0.16	1.77	1.52	18.6	20

**Table 4:** Significance of relationship between fixation times in seconds distinguished by first-point gaze, longest gaze during the test and overall observation time for each image without clear aligners worn.

	*t*-Test	*p*	Meaning
First-point gaze (comparison between groups)	−0.37731	0.356915	Not significant
Longest gaze (comparison between groups)	1.27362	0.115803	Not significant
Overall observation time (comparison between groups)	0.08133	0.468391	Not significant
First-point gaze–Longest gaze comparison in A group	−11.54622	<0.00001	Significant
First-point gaze–Longest gaze comparison in B group	−18.32856	<0.00001	Significant
First-point gaze–overall observation time in A group	−22.47117	<0.00001	Significant
First-point gaze–overall observation time in B group	−14.6492	<0.00001	Significant
Longest gaze–overall observation time comparison in A group	−20.57699	<0.00001	Significant
Longest gaze–overall observation time comparison in B group	−13.67807	<0.00001	Significant

**Table 5:** Significance of relationship between fixation times in seconds distinguished by first-point gaze, longest gaze during the test and overall observation time for each image with clear aligners worn.

	*t*-Test	*p*	Meaning
First-point gaze (comparison between groups)	1.567863	0.1777	Not significant
Longest gaze (comparison between groups)	−1.317402	0.24484	Not significant
Overall observation time (comparison between groups)	−1.438779	0.20974	Not significant
First-point gaze–Longest gaze comparison in A group	13.291117	0.00004	Significant
First-point gaze–Longest gaze comparison in B group	13.453603	0.00004	Significant
First-point gaze–overall observation time in A group	11.708622	0.00008	Significant
First-point gaze–overall observation time in B group	17.995506	<0.00001	Significant
Longest gaze–overall observation time comparison in A group	10.536119	0.00013	Significant
Longest gaze–overall observation time comparison in B group	16.270141	0.00002	Significant

**Table 6:** Significance of relationship between fixation times in seconds distinguished by first-point gaze, longest gaze during the test and overall observation time for both the subgroups of images (without and with clear aligners worn) in the two groups of subjects (A and B group).

	*t*-Test	*p*	Meaning
First-point gaze in A group (comparison between images)	−2.84503	0.008699	Significant
First-point gaze in B group (comparison between images)	−1.96373	0.038976	Significant
Longest gaze in A group (comparison between images)	−0.90427	0.193559	Not significant
Longest gaze in B group (comparison between images)	−2.07183	0.032542	Significant
Overall observation time in A group (comparison between images)	−1.99944	0.036728	Significant
Overall observation time in B group (comparison between images)	−1.74829	0.055493	Not significant

**Table 7:** Rating in stars for each image (mean values) without clear aligners worn (control images).

Rating in Stars	Group A	Group B
Smile 1	3	2
Smile 2	2.5	2.5
Smile 3	3.5	4
Smile 4	3.5	3.5
Smile 5	3	3
Smile 6	4	4.5

**Table 8:** Rating in stars for each image (mean values) wearing clear aligners.

Rating in Stars	Group A	Group B
Smile 1	2	1.5
Smile 2	3	2.5
Smile 3	4.5	4.5
Smile 4	3	3
Smile 5	3	2
Smile 6	5	5

**Table 9:** Significance of comparison between the two groups based on rating in star scores and images and correlation between different gaze times (*p* < 0.05).

	Mann–Whitney U Test	*p*	Meaning
Rating in stars comparison between groups for images without clear aligners worn	U = 18	5	Not significant
Rating in stars comparison between groups for images with clear aligners worn	U = 14	5	Not significant
Rating in stars comparison between images with and without clear aligners worn in group A	U = 18	5	Not significant
Rating in stars comparison between images with and without clear aligners worn in group B	U = 16	5	Not significant
	Spearman’s rho test (*rs*)		
Rating in stars comparison with first-point gaze in group A for images without clear aligners worn	−0.2354	0.65343	Not significant
Rating in stars comparison with first-point gaze in group A for images with clear aligners worn	−0.63754	0.17326	Not sig-nificant
Rating in stars comparison with first-point gaze in group B for images without clear aligners worn	−0.54286	0.2657	Not significant
Rating in stars comparison with first-point gaze in group B for images with clear aligners worn	−0.48571	0.32872	Not significant
Rating in stars comparison with longest gaze in group A for images without clear aligners worn	−0.02942	0.95588	Not significant
Rating in stars comparison with longest gaze in group A for images with clear aligners worn	0.5161	0.29458	Not significant
Rating in stars comparison with longest gaze in group B for images without clear aligners worn	0.25714	0.62279	Not significant
Rating in stars comparison with longest gaze in group B for images with clear aligners worn	0.2	0.704	Not significant
Rating in stars comparison with overall observation time in group A for images without clear aligners worn	−0.47079	0.34599	Not significant
Rating in stars comparison with overall observation time in group A for images with clear aligners worn	−0.94112	0.0051	Significant
Rating in stars comparison with overall observation time in group B for images without clear aligners worn	−0.77143	0.0724	Not sig-nificant
Rating in stars comparison with overall observation time in group B for images with clear aligners worn	−0.88571	0.01885	Significant

**Table 10:** Significance of the influence between overall observation time and rating in stars with the presence of lips and cheek retractors based on clear aligners worn or not worn in the images examined (*p* < 0.05).

	W Walue	Mean Difference	z Value	Sample Size N	Meaning
Overall observation time related to presence of lips/cheek retractors in both groups for images without clear aligners worn	10	4.62	−0.1048	5	Not significant
Overall observation time related to presence of lips/cheek retractors in both groups for images with clear aligners worn	1	−0.15	−1.299917	6	Significant
Rating in stars related to the presence of lips/cheek retractors in both groups for images without clear aligners worn	6	−0.42	0.9435	6	Not significant
Rating in stars related to the presence of lips/cheek retractors in both groups for images with clear aligners worn	0	−0.1	−2.0226	5	Significant
Overall observation time related to presence/absence of clear aligners in both groups	7	−4.7	−2.5103	12	Significant
Overall observation time related to presence/absence of clear aligners and cheek retractors in both groups	1	−5.93	−1.9917	6	Significant
Overall observation time related to presence/absence of clear aligners and lips in both groups	4	−3.67	−1.3628	6	Not significant
Rating in stars related to the presence/absence of clear aligners in both groups	27.5	0.3	0	10	Not significant
Rating in stars related to the presence/absence of clear aligners and cheek retractors in both groups	5.5	1.9	−0.5394	5	Not significant
Rating in stars related to the presence/absence of clear aligners and lips in both groups	5	1.1	−0.6742	5	Not significant

**Table 11:** Distribution by areas of interest at the first-point gaze and at the longest point gaze in both groups of respondents (groups A and B) for each image examined (NV = not valuable).

First-Point Gaze (Area of Interest)							
GROUP A	Anterior Attachments	Posterior Attachments	Gingival Margin	Upper Lip	Lower Lip	Anterior Teeth	Posterior Teeth
Smile 1—no clear aligners	51%	30%	14%	NV	NV	5%	NV
Smile 1—with clear aligners	63%	33%	2%	NV	NV	2%	NV
Smile 2—no clear aligners	NV	16%	3%	30%	41%	10%	NV
Smile 2—with clear aligners	NV	19%	1%	26%	52%	3%	NV
Smile 3—no clear aligners	NV	NV	37%	NV	NV	45%	18%
Smile 3—with clear aligners	NV	NV	34%	NV	NV	37%	29%
Smile 4—no clear aligners	37%	14%	5%	16%	20%	8%	NV
Smile 4—with clear aligners	31%	22%	2%	22%	18%	5%	NV
Smile 5—no clear aligners	NV	50%	11%	NV	NV	39%	NV
Smile 5—with clear aligners	NV	46%	13%	NV	NV	41%	NV
Smile 6—no clear aligners	NV	NV	18%	30%	28%	18%	8%
Smile 6—with clear aligners	NV	NV	11%	32%	24%	20%	13%
GROUP B	Anterior attachments	Posterior attachments	Gingival margin	Upper Lip	Lower Lip	Anterior teeth	Posterior teeth
Smile 1—no clear aligners	57%	30%	8%	NV	NV	5%	NV
Smile 1—with clear aligners	55%	39%	4%	NV	NV	2%	NV
Smile 2—no clear aligners	NV	23%	3%	28%	30%	16%	NV
Smile 2—with clear aligners	NV	27%	3%	29%	23%	12%	NV
Smile 3—no clear aligners	NV	NV	48%	NV	NV	42%	10%
Smile 3—with clear aligners	NV	NV	41%	NV	NV	38%	21%
Smile 4—no clear aligners	29%	28%	5%	23%	11%	4%	NV
Smile 4—with clear aligners	33%	26%	3%	23%	9%	6%	NV
Smile 5—no clear aligners	NV	35%	24%	NV	NV	41%	NV
Smile 5—with clear aligners	NV	39%	21%	NV	NV	40%	NV
Smile 6—no clear aligners	NV	NV	11%	26%	31%	24%	8%
Smile 6—with clear aligners	NV	NV	9%	27%	29%	27%	8%
Longest gaze (area of interest)							
GROUP A	Anterior attachments	Posterior attachments	Gingival margin	Upper Lip	Lower Lip	Anterior teeth	Posterior teeth
Smile 1—no clear aligners	56%	40%	2%	NV	NV	2%	NV
Smile 1—with clear aligners	52%	41%	3%	NV	NV	4%	NV
Smile 2—no clear aligners	NV	31%	5%	30%	24%	10%	NV
Smile 2—with clear aligners	NV	26%	8%	36%	22%	8%	NV
Smile 3—no clear aligners	NV	NV	28%	NV	NV	40%	32%
Smile 3—with clear aligners	NV	NV	21%	NV	NV	33%	46%
Smile 4—no clear aligners	30%	28%	5%	18%	11%	8%	NV
Smile 4—with clear aligners	26%	24%	1%	20%	22%	7%	NV
Smile 5—no clear aligners	NV	60%	27%	NV	NV	13%	NV
Smile 5—with clear aligners	NV	64%	24%	NV	NV	12%	NV
Smile 6—no clear aligners	NV	NV	20%	42%	28%	7	3%
Smile 6—with clear aligners	NV	NV	26%	38%	26%	9%	1%
GROUP B	Anterior attachments	Posterior attachments	Gingival margin	Upper Lip	Lower Lip	Anterior teeth	Posterior teeth
Smile 1—no clear aligners	54%	38%	5%	NV	NV	3%	NV
Smile 1—with clear aligners	58%	37%	3%	NV	NV	2%	NV
Smile 2—no clear aligners	NV	32%	4%	36%	18%	10%	NV
Smile 2—with clear aligners	NV	29%	6%	33%	19%	13%	NV
Smile 3—no clear aligners	NV	NV	42%	NV	NV	38%	20%
Smile 3—with clear aligners	NV	NV	40%	NV	NV	34%	26%
Smile 4—no clear aligners	31%	35%	4%	12%	8%	10%	NV
Smile 4—with clear aligners	34%	37%	1%	15%	7%	6%	NV
Smile 5—no clear aligners	NV	41%	14%	NV	NV	45%	NV
Smile 5—with clear aligners	NV	42%	18%	NV	NV	40%	NV
Smile 6—no clear aligners	NV	NV	13%	29%	22%	29%	7%
Smile 6—with clear aligners	NV	NV	12%	27%	20%	31%	10%

**Table 12:** Comparison between the two groups based on the first-point gaze and longest point gaze in the different areas of interest in the images *without* clear aligners worn (*p* < 0.05).

	Chi-Square Test	*p*-Value
Smile 1 first-point gaze (area of interest)—comparison between A and B groups	1.9697	0.57872
Smile 2 first-point gaze (area of interest)—comparison between A and B groups	4.4142	0.352841
Smile 3 first-point gaze (area of interest)—comparison between A and B groups	3.8127	0.148622
Smile 4 first-point gaze (area of interest)—comparison between A and B groups	10.839	0.05466725
Smile 5 first-point gaze (area of interest)—comparison between A and B groups	7.5256	0.023218
Smile 6 first-point gaze (area of interest)—comparison between A and B groups	3.7279	0.444079
Smile 1 longest gaze (area of interest)—comparison between A and B groups	1.5734	0.665445
Smile 2 longest gaze (area of interest)—comparison between A and B groups	1.5296	0.821389
Smile 3 longest gaze (area of interest)—comparison between A and B groups	5.6205	0.06019
Smile 4 longest gaze (area of interest)—comparison between A and B groups	2.801	0.73063284
Smile 5 longest gaze (area of interest)—comparison between A and B groups	25.3514	<0.00001
Smile 6 longest gaze (area of interest)—comparison between A and B groups	19.6296	0.000591

**Table 13:** Comparison between the two groups based on the first-point gaze and longest point gaze in the different areas of interest in the images with clear aligners worn (*p* < 0.05).

	Chi-Square Test	*p*-Value
Smile 1 first-point gaze (area of interest)—comparison between A and B groups	2.0374	0.564681
Smile 2 first-point gaze (area of interest)—comparison between A and B groups	19.8667	0.000531
Smile 3 first-point gaze (area of interest)—comparison between A and B groups	1.9474	0.37768
Smile 4 first-point gaze (area of interest)—comparison between A and B groups	4.085	0.53724414
Smile 5 first-point gaze (area of interest)—comparison between A and B groups	9.7966	0.007459
Smile 6 first-point gaze (area of interest)—comparison between A and B groups	2.6045	0.626034
Smile 1 longest gaze (area of interest)—comparison between A and B groups	1.0921	0.778988
Smile 2 longest gaze (area of interest)—comparison between A and B groups	1.9898	0.73764
Smile 3 longest gaze (area of interest)—comparison between A and B groups	11.4885	0.003201
Smile 4 longest gaze (area of interest)—comparison between A and B groups	13.093	0.02252267
Smile 5 longest gaze (area of interest)—comparison between A and B groups	20.5001	0.000035
Smile 6 longest gaze (area of interest)—comparison between A and B groups	27.2657	0.000018

**Table 14:** Comparison between images without and with clear aligners worn based on the first-point gaze and longest point gaze in the different areas of interest in the smiles (*p* < 0.05).

	Chi-Square Test	*p*-Value
Smile 1 first-point gaze (area of interest)—comparison between image without and with clear aligners	17.5352	0.040965
Smile 2 first-point gaze (area of interest)—comparison between image without and with clear aligners	26.348	0.00958
Smile 3 first-point gaze (area of interest)—comparison between image without and with clear aligners	13.2495	0.03924
Smile 4 first-point gaze (area of interest)—comparison between image without and with clear aligners	16.991	0.31940271
Smile 5 first-point gaze (area of interest)—comparison between image without and with clear aligners	10.06	0.122149
Smile 6 first-point gaze (area of interest)—comparison between image without and with clear aligners	10.7241	0.552695
Smile 1 longest gaze (area of interest)—comparison between image without and with clear aligners	3.0816	0.960972
Smile 2 longest gaze (area of interest)—comparison between image without and with clear aligners	5.3072	0.946922
Smile 3 longest gaze (area of interest)—comparison between image without and with clear aligners	22.0256	0.001198
Smile 4 longest gaze (area of interest)—comparison between image without and with clear aligners	26.08	0.03719016
Smile 5 longest gaze (area of interest)—comparison between image without and with clear aligners	46.4368	<0.00001
Smile 6 longest gaze (area of interest)—comparison between image without and with clear aligners	48.4195	<0.00001

**Table 15:** Relationship between intra-group variability and inter-group variability when comparing images of smiles with and without aligners worn on the basis of first gaze point.

Source	Sum of Squares SS	Degrees of Freedom νν	Mean Square MS	F-Statistic	*p*-Value
Between	3968.5429	5	793.7086	4.1751	0.0018
Within	18,630.2167	98	190.1043		
Total	22,598.7596	103			

**Table 16:** Relationship between intra-group variability and inter-group variability when comparing images of smiles with and without aligners worn on the basis of longest gaze point.

Source	Sum of Squares SS	Degrees of Freedom νν	Mean Square MS	F-Statistic	*p*-Value
Between	3948.7179	5	789.7436	3.7366	0.0039
Within	20,712.667	98	211.3537		
Total	24,661.3846	103			

**Table 17:** Post-hoc tests applied to comparison pairs of smiles with and without clear aligners worn based on first-point gaze. Values in bold with asterisk represent significantly different comparisons.

Comparison Pair	Tukey HSD Q Statistic	Tukey HSD *p*-Value	Scheffè T-Statistic	Scheffè *p*-Value	Bonferroni and Holm T-Statistic	Bonferroni *p*-Value	Holm *p*-Value
Smile 1 vs. Smile 2	1.6055	0.8517601	1.1352	0.9349495	1.1352	3.8856641	1.5542657
Smile 1 vs. Smile 3	2.2383	0.5970256	1.5827	0.7748420	1.5827	1.7507304	0.9337229
Smile 1 vs. Smile 4	2.6483	0.4269844	1.8727	0.6237152	1.8727	0.9614499	0.5768699
Smile 1 vs. Smile 5	2.2383	0.5970256	1.5827	0.7748420	1.5827	1.7507304	0.8170075
Smile 1 vs. Smile 6	1.4984	0.8948455	1.0596	0.9511189	1.0596	4.3792674	1.4597558
Smile 2 vs. Smile 3	3.8155	0.0850264	2.6980	0.2113136	2.6980	0.1232246	0.1067946
Smile 2 vs. Smile 4	1.0446	0.8999947	0.7386	0.9900823	0.7386	6.9285801	1.3857160
Smile 2 vs. Smile 5	3.8155	0.0850264	2.6980	0.2113136	2.6980	0.1232246	0.0985797
Smile 2 vs. Smile 6	0.1135	0.8999947	0.0803	0.9999998	0.0803	14.0427517	1.8723669
Smile 3 vs. Smile 4	4.8352	0.0114955 *	3.4190	0.0473774 *	3.4190	0.0137588 *	0.0137588 *
Smile 3 vs. Smile 5	0.0000	0.8999947	0.0000	1.0000000	0.0000	15.0000000	1.0000000
Smile 3 vs. Smile 6	3.7172	0.1000928	2.6285	0.2377420	2.6285	0.1493660	0.1095351
Smile 4 vs. Smile 5	4.8352	0.0114955 *	3.4190	0.0473774 *	3.4190	0.0137588 *	0.0128415 *
Smile 4 vs. Smile 6	1.1631	0.8999947	0.8225	0.9838156	0.8225	6.1921848	1.6512493
Smile 5 vs. Smile 6	3.7172	0.1000928	2.6285	0.2377420	2.6285	0.1493660	0.0995774

**Table 18:** Post-hoc tests applied to comparison pairs of smiles with and without clear aligners worn based on longest point gaze. Values in bold with asterisk represent significantly different comparisons.

Comparison Pair	Tukey HSD Q Statistic	Tukey HSD *p*-Value	Scheffè T-Statistic	Scheffè *p*-Value	Bonferroni and Holm T-Statistic	Bonferroni *p*-Value	Holm *p*-Value
Smile 1 vs. Smile 2	1.4501	0.8999947	1.0254	0.9574354	1.0254	4.6155589	1.8462236
Smile 1 vs. Smile 3	2.1228	0.6435194	1.5010	0.8119233	1.5010	2.0484975	1.0925320
Smile 1 vs. Smile 4	2.5117	0.4862373	1.7760	0.6766135	1.7760	1.1824840	0.7094904
Smile 1 vs. Smile 5	2.1228	0.6435194	1.5010	0.8119233	1.5010	2.0484975	0.9559655
Smile 1 vs. Smile 6	1.4501	0.8999947	1.0254	0.9574354	1.0254	4.6155589	1.5385196
Smile 2 vs. Smile 3	3.5521	0.1304538	2.5117	0.2866552	2.5117	0.2047503	0.1774502
Smile 2 vs. Smile 4	1.0710	0.8999947	0.7573	0.9888774	0.7573	6.7603059	1.8027482
Smile 2 vs. Smile 5	3.5521	0.1304538	2.5117	0.2866552	2.5117	0.2047503	0.1638002
Smile 2 vs. Smile 6	0.0000	0.8999947	0.0000	1.0000000	0.0000	15.0000000	2.0000000
Smile 3 vs. Smile 4	4.5857	0.0195893 *	3.2426	0.0714119	3.2426	0.0242962 *	0.0242962 *
Smile 3 vs. Smile 5	0.0000	0.8999947	0.0000	1.0000000	0.0000	15.0000000	1.0000000
Smile 3 vs. Smile 6	3.5521	0.1304538	2.5117	0.2866552	2.5117	0.2047503	0.1501502
Smile 4 vs. Smile 5	4.5857	0.0195893 *	3.2426	0.0714119	3.2426	0.0242962 *	0.0226765 *
Smile 4 vs. Smile 6	1.0710	0.8999947	0.7573	0.9888774	0.7573	6.7603059	1.3520612
Smile 5 vs. Smile 6	3.5521	0.1304538	2.5117	0.2866552	2.5117	0.2047503	0.1365002

## Data Availability

Data is available on request.
